# Pharmacogenetic CYP2D6 variability, phenoconversion and treatment outcomes: A Danish population-based cohort study in 6,798 individuals initiating atomoxetine treatment

**DOI:** 10.1192/j.eurpsy.2022.588

**Published:** 2022-09-01

**Authors:** K. Ishtiak-Ahmed, C. Lunenburg, J. Thirstrup, L. Clausen, P. Thomsen, C. Gasse

**Affiliations:** 1 Aarhus University Hospital Psychiatry, Department Of Affective Disorders, Aarhus N, Denmark; 2 Aarhus University, Department Of Clinical Medicine, Aarhus N, Denmark; 3 Aarhus University Hospital Psychiatry, Department Of Child & Adolescent Psychiatry, Aarhus N, Denmark

**Keywords:** treatment switching, CYP2D6 inhibitors, adhd, sleeping problem

## Abstract

**Introduction:**

Atomoxetine, a first-line treatment option for ADHD, is affected by pharmacogenetic (PGx) variation of the drug-metabolizing enzyme CYP2D6. Despite the recommendation of CYP2D6 testing, the use of PGx-guided dosing remains low in Denmark.

**Objectives:**

We investigated wide-ranging clinical outcomes in atomoxetine users in association with PGx variability.

**Methods:**

We analyzed 6,798 individuals (55% children) with a first prescription for atomoxetine identified from the Danish population-based iPSYCH case-cohort study linking biobank information with Danish registers. Individuals were categorized based on their single-nucleotide-polymorphism-based CYP2D6 genotype into normal (NM), intermediate (IM), and poor metabolizer (PM). Clinical outcomes included treatment switching, discontinuation, psychiatric inpatient-, outpatient-, and emergency contact, suicide attempt/self-harm, sleep problem, and depression. Individuals’ CYP2D6-status could change due to drug-drug-interactions of weak, intermediate, or strong-CYP2D6 inhibitors (phenoconversion), which we accounted for by time-varying phenotype assessment. Incidence rate ratios (IRR) were estimated using Poisson regression analyses and adjusted for a wide range of potential confounders and covariates.

**Results:**

Over two-thirds of the individuals had a hospital diagnosis of ADHD at the first atomoxetine prescription. The distribution of CYP2D6 phenotypes was similar in children and adults. IM/PM children had a significantly higher risk of a sleeping problem compared with NM children (IRR 1.25, 95% CI 1.01-1.54). Compared with NM adults, those with IM/PM had a higher risk of switching (1.15, 95% CI 0.98-1.35).

**Conclusions:**

This is the first study showing the potential impact of PGx variability on clinical outcomes of atomoxetine users in a population-based setting, highlighting the utility of PGx testing.

**Disclosure:**

No significant relationships.

